# Korean Risk Assessment Model for Breast Cancer Risk Prediction

**DOI:** 10.1371/journal.pone.0076736

**Published:** 2013-10-25

**Authors:** Boyoung Park, Seung Hyun Ma, Aesun Shin, Myung-Chul Chang, Ji-Yeob Choi, Sungwan Kim, Wonshik Han, Dong-Young Noh, Sei-Hyun Ahn, Daehee Kang, Keun-Young Yoo, Sue K. Park

**Affiliations:** 1 National Cancer Control Institute, National Cancer Center, Goyang, Korea; 2 Department of Preventive Medicine, Seoul National University College of Medicine, Seoul, Korea; 3 Department of Biomedical Science, Seoul National University Graduate School, Seoul, Korea; 4 Cancer Research Institute, Seoul National University, Seoul, Korea; 5 Molecular Epidemiology Branch, Research Institute, National Cancer Center, Goyang, Korea; 6 Department of Surgery, Dankook University Hospital, Seoul, Korea; 7 Department of Biomedical Engineering, Seoul National University College of Medicine, Seoul, Korea; 8 Institute of Medical and Biological Engineering, Medical Research Center, Seoul National University, Seoul, Korea; 9 Department of Surgery, Seoul National University Hospital and College of Medicine, Seoul, Korea; 10 Department of Surgery, Asan Medical Center, Ulsan University College of Medicine, Seoul, Korea; Dartmouth, United States of America

## Abstract

**Purpose:**

We evaluated the performance of the Gail model for a Korean population and developed a Korean breast cancer risk assessment tool (KoBCRAT) based upon equations developed for the Gail model for predicting breast cancer risk.

**Methods:**

Using 3,789 sets of cases and controls, risk factors for breast cancer among Koreans were identified. Individual probabilities were projected using Gail's equations and Korean hazard data. We compared the 5-year and lifetime risk produced using the modified Gail model which applied Korean incidence and mortality data and the parameter estimators from the original Gail model with those produced using the KoBCRAT. We validated the KoBCRAT based on the expected/observed breast cancer incidence and area under the curve (AUC) using two Korean cohorts: the Korean Multicenter Cancer Cohort (KMCC) and National Cancer Center (NCC) cohort.

**Results:**

The major risk factors under the age of 50 were family history, age at menarche, age at first full-term pregnancy, menopausal status, breastfeeding duration, oral contraceptive usage, and exercise, while those at and over the age of 50 were family history, age at menarche, age at menopause, pregnancy experience, body mass index, oral contraceptive usage, and exercise. The modified Gail model produced lower 5-year risk for the cases than for the controls (*p* = 0.017), while the KoBCRAT produced higher 5-year and lifetime risk for the cases than for the controls (*p*<0.001 and <0.001, respectively). The observed incidence of breast cancer in the two cohorts was similar to the expected incidence from the KoBCRAT (KMCC, *p* = 0.880; NCC, *p* = 0.878). The AUC using the KoBCRAT was 0.61 for the KMCC and 0.89 for the NCC cohort.

**Conclusions:**

Our findings suggest that the KoBCRAT is a better tool for predicting the risk of breast cancer in Korean women, especially urban women.

## Introduction

Breast cancer is the second most common malignancy among women in Korea. The incidence of breast cancer is increasing rapidly, at an average rate second only to that of thyroid cancer. In 2009, 13,399 new female cases of breast cancer developed and 1,878 women died of this disease [1].

Several breast cancer screening modalities are currently available, including clinical breast examination, mammography, breast ultrasonography, and breast magnetic resonance imaging. Biannual mammography is provided as an organized cancer screening program for women who are 40 years and older in Korea. Given the high incidence of breast cancer, its significance, and the various available screening tests, a model estimating an individual's risk of developing breast cancer that could be easily applied in the clinical setting may be useful for recommending supplementary screening tests and conducting chemoprevention or screening intervention trials [2].

Several risk factors for breast cancer have been identified [3], and mathematical models estimating breast cancer risk based on defined risk factors have been developed in Western countries. In Western population, the Gail model is predominantly applied to select high-risk women in the general population, and other models are applied to specific populations, such as those with familiar clusters of breast or ovarian cancers [4]. Based on the results from mathematical models, risk-reduction strategies such as lifestyle modification, chemoprevention, or surgical approaches have been applied in high-risk women [5].

The incidence of breast cancer and various lifestyle or reproductive risk factors differ markedly among ethnic groups [6]. These differences may hinder the applicability of risk assessment models developed in Western countries, such as the Gail model, in Korea.

The objectives of this study were to develop the Korean Breast Cancer Risk Assessment Tool (KoBCRAT) using major risk factors for breast cancer, incidence and mortality rate from Korean data, and Gail's equations for projecting individual breast cancer probability in a Korean population; to evaluate the performance of the Gail model and the KoBCRAT; and to validate the KoBCRAT.

## Materials and Methods

### Study population for selection of major risk factors for breast cancer

This study was based on the Seoul Breast Cancer Study (SeBCS) recruited from 1994 to 2007. The cases (N = 4,601) consisted of women diagnosed with histologically confirmed breast cancer who admitted to three teaching hospitals located in Seoul, accounting for about 15–18% of total breast cancer cases in Korea. The controls (N = 4,647) were composed of non-cancer patients visiting the same hospitals as the cases from 1994 to 1997, health examinees visited to same hospitals from 1998 to 2000, and healthy women without cancer who participated in the community health screening program provided by teaching hospitals located in urban area from 2001 to 2007. After getting written informed consent, information on demographic characteristics, reproductive factors, and lifestyle habits were collected by trained interviewers using a structured questionnaire. The cases and controls were frequency-matched using 5-year age groups (<20, 20–24, 25–29, 30–34, 35–39, 40–44, 45–49, 50–54, 55–59, 60–64, 65–69, and ≥70 years old) and the enrollment year (1994–1997, 1998–2000, and 2001–2007). To select age-matched cases and controls, a random selection method was applied. As a result, controls <30 years old or ≥40 years old and cases in their thirties, when the number of participants was higher than in the other group, were randomly excluded. Ultimately, 3,789 sets of cases and controls were selected for analysis. The distribution of characteristics among the cases and controls were compared using chi-square tests. The study design and the present study were approved by the Seoul National University institutional review board (IRB number: C-0909-048-295).

### Individual risk projection for the KoBCRAT

We assessed a variety of factors that have been consistently associated with breast cancer [3], [7], [8], including family history of breast cancer, age at menarche, menopausal status, age at menopause, experience of pregnancy, age at first full-term pregnancy, number of pregnancies, duration of breastfeeding, oral contraceptive usage, hormone replacement therapy, exercise, body mass index (BMI), smoking, drinking, and number of breast examinations. In the case of variables with missing information, we included an unknown category in the models.

This study used the same statistical approach as in the Gail model [9]. Risk factors included in the model were selected by Wald tests for individual parameters as well as known risk factors for breast cancer. We applied a number of selection methods, including forward selection, backward elimination, and stepwise logistic regression. Odds ratios (ORs) were calculated using unconditional logistic regression models for two age categories (<50 and ≥50 years old). Although conditional logistic regression has generally been applied to matched datasets, we used an unconditional logistic regression model for several reasons. First, Czech's breast cancer risk assessment model, a modified Gail model, uses an unconditional logistic regression despite the inclusion of matched case-control data [10]. Second, we conducted stratification analyses of hormone receptor status, and matching of the subjects was broken after stratification. Additionally, we included an “unknown category” in the model as a tool for predicting the risk of developing colorectal cancer [11]. We conducted both conditional logistic regression and unconditional logistic regression analyses, and the results were similar, showing minimal differences.

Individual probabilities of developing breast cancer were projected by combining information on an individual's relative risk, baseline hazard rate, and competing risk. The breast cancer incidence rate was obtained from Korean National Cancer Registry data and the total mortality rate and breast cancer specific mortality rate were obtained from Statistics of deaths by cause from the Korea National Statistical Office (Appendix 1). We measured the discriminatory accuracy of the model using the area under the receiver operating curve (AUC). All statistical analyses were performed using SAS software (version 9.1; SAS Institute Inc., Cary, NC, USA) and Microsoft Excel 2007 (Microsoft Inc., Redmond, WA, USA).

### Evaluation of the KoBCRAT performance

To compare the performance of the KoBCRAT with that of the Gail model, we calculated and compared the mean risk for 3,789 age-matched case-control pairs from the SeBCS using parameter estimators as in the original Gail model, as well as Korean incidence and mortality data. Also, we compared the mean risk for 3,789 age-matched case-control pairs using the KoBCRAT. We also validated the KoBCRAT in two large, independent, prospective Korean cohort studies with incident breast cancer cases: the Korean Multi-Center Cohort (KMCC) study, a community general population cohort recruited from four rural regions [12], [13], and the National Cancer Center (NCC) cohort study, with subjects recruited from the NCC cancer screening program in metropolitan city [14]. To ascertain new cancer cases, the KMCC and NCC cohort study adopted a passive follow-up system using record linkage with the Korea Central Cancer Registry and the National Death Certification databases. As of December 2009, 29 and 24 breast cancer cases were ascertained among 11,905 KMCC and 9,664 NCC female cohort participants, respectively.

### Statistical analysis for performance evaluation

We calculated the means and standard deviations of 5-year and lifetime breast cancer risks up to the age of 90 years old in breast cancer case-control sets using the KoBCRAT, the Gail model, and the modified Gail model and compared the differences in mean values between cases and controls from each model by T- test.

In the KMCC and the NCC cohort, we compared the expected and observed numbers of incident breast cancer cases overall. The expected number of breast cancer cases was calculated by adding the individual absolute risk for each person calculated by the KoBCRAT and compared using a chi-squared test. The ratio of expected to observed numbers of cases (E/O ratio) and its 95% CI (confidence interval) were calculated with the following equation [15]:
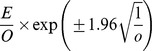
All statistical analyses were performed using SAS software (version 9.1; SAS Institute Inc.).

## Results

The mean age of the 3,789 cases and 3,789 controls was 49.0±9.47 years. Details about the characteristics of cases and controls were presented and compared in Appendix 2. The same variables as risk factors of breast cancer were included in the model when each of forward selection, backward elimination, and stepwise regression was applied. The risk factors and their levels and corresponding ORs are given in Table 1. For women aged <50 years, a family history of breast cancer in first-degree relatives, age at menarche, menopausal status, age at first full-term pregnancy, duration of breast feeding, oral contraceptive usage, and exercise are included. A family history of breast cancer in first-degree relatives, age at menarche, age at menopause, experience of pregnancy, BMI, oral contraceptive usage, and exercise are included in the final model among women aged ≥50 years. The natural logarithms of ORs and their corresponding risk factors for risk calculation are shown in the Appendix 3. The estimated baseline hazard increased with age, peaking in the 45–49-year age group (43.1/100,000 women-years) and then declining (Appendix 1).

**Table 1:** Relative risks of major risk factors for breast cancer in Korean women.

	Age <50 years		Age ≥50 years	
	OR^a^	(95% CI^b^)	OR	(95% CI)
**Family history of breast cancer in first-degree relatives**				
No	1		1	
Yes	1.12	(0.81–1.56)	2.01	(1.28–3.13)
**Age at menarche (years)**				
<13	1.87	(1.37–2.54)	2.40	(1.38–4.19)
13–16	1.44	(1.17–1.76)	1.53	(1.29–1.81)
≥17	1		1	
**Menopause**				
Premenopausal	1.74	(1.42–2.14)		
Postmenopausal	1			
**Age at menopause (years)**				
Premenopausal			2.50	(1.78–3.51)
<44			1	
45–49			1.34	(0.99–1.83)
50–54			1.36	(1.01–1.82)
≥55			1.62	(1.09–2.39)
**Pregnancy**				
Nullipara			1.88	(1.24–2.84)
Para			1	
**Age at first full-term pregnancy (years)**				
Nullipara	1.08	(0.80–1.45)		
<24	1			
24–30	1.16	(0.97–1.39)		
≥31	1.25	(0.93–1.69)		
**Duration of Breast feeding (months)**				
Never	0.93	(0.77–1.12)		
0∼6	1.25	(1.01–1.53)		
>6	1			
**Body mass index**				
<25			1	
25–29.9			1.16	(0.97–1.38)
≥30			2.28	(1.49–3.48)
**Oral contraceptive usage**				
Never	1		1	
Ever	1.24	(0.91–1.69)	1.52	(1.12–2.06)
**Exercise**				
<once/week	1.33	(1.12–1.59)	1.84	(1.50–2.27)
≥once/week	1		1	

a Odds ratio.

b confidence interval.

Table 2 shows estimated absolute risks according to various initial relative risks, relative risks at the age of 50 years, initial age, and follow-up periods. When the risk projection interval exceeded 50 years of age, initial relative risk and relative risk at the age of 50 years were applied. A 30-year-old woman with the highest risk at ages 30 and 50 years showed a nearly 22-fold greater risk after 30 years and a 26-fold greater risk at 90 years of age compared with those with minimal risk. The maximum values of lifetime breast cancer risk up to the age of 90 years were 57.2% in women aged 30 years and 44.4% in women aged 50 years. The discriminatory powers measured by AUCs of the KoBCRAT were 0.63 (95% CI, 0.61–0.65) for women <50 years old and 0.65 (95% CI, 0.61–0.68) for women ≥50 years old (data not shown).

**Table 2:** Estimated absolute risk (%) of developing breast cancer in Korean women by initial and later relative risks, initial age, and years of follow-up.

Initial age (years)	Follow-up (years)	Later relative risk^b^	Initial relative risk^a^					
			1	2	5	10	-	
30	10		0.18	0.35	0.88	1.74	-	
	20		1.67	3.31	8.07	15.49	-	
	30	1	1.96	3.59	8.34	15.73	-	
		10	4.51	6.10	10.72	17.91	-	
		20	7.27	8.81	13.29	20.26	-	
		50	15.05	16.46	20.54	26.89		
		100	26.54	27.74	31.23	36.67	-	
		144	35.27	36.32	39.37	44.11	-	
	40	1	2.09	3.72	8.46	15.84	-	
		10	5.75	7.32	11.88	18.97	-	
		20	9.66	11.16	15.51	22.29	-	
		50	20.38	21.69	25.50	31.43		
		100	35.35	36.39	39.43	44.18	-	
		144	46.00	46.86	49.36	53.25	-	
	50	1	2.14	3.77	8.51	15.89	-	
		10	6.27	7.83	12.36	19.41	-	
		20	10.64	12.12	16.43	23.13	-	
		50	22.49	23.77	27.46	33.22		
		100	38.62	39.61	42.48	46.97	-	
		144	49.78	50.57	52.87	56.47	-	
	60	1	2.15	3.79	8.52	15.90	-	
		10	6.39	7.95	12.47	19.51	-	
		20	10.88	12.36	16.65	23.33	-	
		50	22.99	24.26	27.93	33.65		
		100	39.38	40.35	43.19	47.61	-	
		144	50.63	51.41	53.67	57.20	-	
40	10		0.42	0.83	2.07	4.11	-	
	20	1	0.71	1.13	2.36	4.39	-	
		10	3.32	3.72	4.92	6.89	-	
		20	6.13	6.52	7.69	9.60		
		50	14.07	14.43	15.49	17.23	-	
		100	25.78	26.09	27.00	28.49	-	
		144	34.69	34.96	35.75	37.06	-	
	30	1	0.84	1.26	2.49	4.51	-	
		10	4.58	4.98	6.17	8.11	-	
		20	8.57	8.95	10.08	11.94	-	
		50	19.50	19.84	20.83	22.45	-	
		100	34.77	35.03	35.83	37.13		
		144	45.64	45.86	46.51	47.58	-	
	40	1	0.90	1.31	2.55	4.57	-	
		10	5.11	5.51	6.68	8.62	-	
		20	9.57	9.94	11.07	12.90	-	
		50	21.66	21.98	22.94	24.52		
		100	0.42	0.83	2.07	4.11	-	
		144	0.71	1.13	2.36	4.39	-	
	50	1	3.32	3.72	4.92	6.89	-	
		10	6.13	6.52	7.69	9.60	-	
		20	14.07	14.43	15.49	17.23		
		50	25.78	26.09	27.00	28.49	-	
		100	38.11	38.36	39.11	40.34	-	
		144	49.49	49.69	50.29	51.28	-	

Initial age (years)	Follow-up year		1	10	20	50	100	144
50	10		0.25	2.44	4.82	11.61	21.85	29.84
	20		0.36	3.51	6.90	16.33	29.90	39.94
	30		0.40	3.96	7.75	18.22	32.97	43.60
	40		0.41	4.07	7.96	18.67	33.68	44.44
60	10		0.14	1.39	2.75	6.70	12.83	17.79
	20		0.20	1.97	3.88	9.35	17.58	24.02
	30		0.21	2.10	4.15	9.97	18.68	25.43

a The initial relative risk corresponds to the initial age a.

b Later relative risk refers to relative risk at age 50 years for a women who was initially younger than age 50 years. For a woman aged 50 years or later initially, no later relative risk is needed. No later relative risk is needed unless the age at the end of follow-up exceeds 50 years.

The performance of the Gail model compared with the KoBCRAT in our 3,789 cases and 3,789 controls is presented in Table 3. When we calculated the 5-year and lifetime risk using the modified Gail model with Korean incidence and mortality data and the parameter estimators from the original Gail model, the 5-year risk was significantly higher for the controls than for the cases (5-year risk: 0.442 for the cases and 0.450 for the controls, *p* = 0.017). With the KoBCRAT, the 5-year and lifetime risk scores were significantly higher for the cases than for the controls, indicating good performance. (5-year risk, 0.48% for cases and 0.40% for controls, *p*<0.001; lifetime risks, 3.00% for cases and 2.61% for controls, *p*<0.001).

**Table 3:** Comparison of 5-year and lifetime risks in a Korean population using the Korean Breast Cancer Risk Assessment Tool (KoBCRAT) and the modified Gail model.

	Case	Control	*p*
	Mean (SD^a^)	Mean (SD^a^)	
Modified Gail model^b^			
5-year risk	0.442 (0.148)	0.450 (0.142)	0.017
Lifetime risk to age 90 years	2.241 (0.957)	2.266 (0.941)	0.258
KoBCRAT			
5-year risk	0.477 (0.348)	0.397 (0.276)	<0.001
Lifetime risk to age 90 years	2.997 (3.670)	2.612 (3.618)	<0.001

a Standard deviation.

b Gail model using Korean 2009 mortality rate and 2008 breast cancer incidence rate.

**Figure 1:**
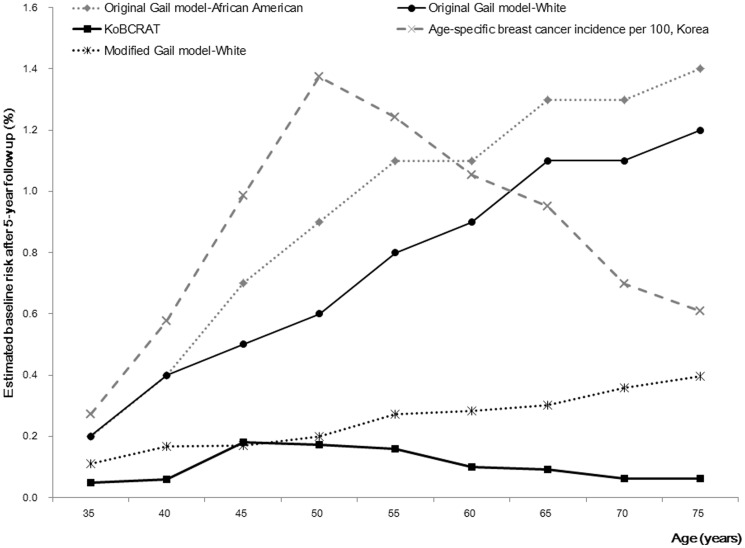
Comparison of the estimated baseline breast cancer risk according to age and 5-year follow-up calculated by the original Gail model, modified Gail model which used parameter estimators as in the original Gail and Korean incidence and mortality data, and Korean Breast Cancer Risk Assessment Tool (KoBCRAT).

Figure 1 shows the baseline breast cancer risk according to age and 5-year follow-up in the three models: the original Gail model (using mortality and incidence data from the US and Gail's parameter estimators), modified Gail model (using Korean mortality and incidence data and Gail's parameter estimators), and the KoBCRAT. There was a marked difference in the baseline risk between the original Gail and KoBCRAT models. The baseline risk in the original Gail model was increased until 80–85 years of age and was much higher than the age-specific breast cancer incidence rate after 60 years of age, while the risk according to the KoBCRAT increased until 45–49 years of age and then decreased, reflecting the trend in age-specific breast cancer incidence in Korea. The modified Gail model showed an increasing baseline risk until 80–85 years of age, although the risks were much lower than in the original Gail model, reflecting the lower incidence and mortality rate of breast cancer in Korea.

We validated the KoBCRAT model using the two Korean cohort studies such as KMCC and the NCC cohort (Table 4). The overall E/O ratio was 0.97 (95% CI, 0.67–1.40) in the KMCC and 0.96 (95% CI, 0.70–1.37) in the NCC cohort. The *p*-values of expected and observed numbers of cases obtained by the chi-squared test were not significant, showing good model calibration (KMCC, *p* = 0.880; NCC, *p* = 0.878). The discriminatory power measured by AUC was 0.61 (95% CI, 0.49–0.72) in the KMCC female participants and 0.89 (95% CI, 0.85–0.93) in the female participants of the NCC cohort.

**Table 4:** Prediction of the number of incident breast cancer cases during the follow-up in two cohort studies (Korean Multi-Center Cohort (KMCC) and National Cancer Center (NCC) cohort) using the Korean Breast Cancer Risk Assessment Tool (KoBCRAT).

	Incident breast cancer cases	Non-breast cancer subjects	*p*
**KMCC**			
	**N**	**N**	
Observed number (O)	29	6119	0.880
Expected number (E)	28.23	6199.77	
E/O ratio	0.97 (95% CI^a^, 0.67–1.40)		
**NCC cohort**			
	**N**	**N**	
Observed number (O)	36	7510	0.878
Expected number (E)	34.71	7511.29	
E/O ratio	0.96(95% CI^a^, 0.70–1.37)		

a Confidence interval.

## Discussion

We established the KoBCRAT using the Gail's equation. The risk factors for breast cancer in the KoBCRAT were selected according to the age categories of <50 and ≥50 year. These risk factors were a family history of breast cancer in the first-degree relatives, age at menarche, menopausal status, age at first full-term pregnancy, duration of breast feeding, oral contraceptive usage, and exercise for women aged <50 years. Risk factors for women aged ≥50 years were a family history of breast cancer in the first-degree relatives, age at menarche, age at menopause, experience of pregnancy, BMI, oral contraceptive usage, and exercise. The Gail model over-predicted the risk of non-cases compared with breast cancer cases for Koreans, whereas the KoBCRAT showed good performance. Estimated numbers of incident breast cancer measured by the KoBCRAT agreed well with the observed numbers in two independent cohorts, showing good validity.

Although the Gail model selected the same risk factors across all age groups, the risks were calculated separately for two age ranges: <50 years and ≥50 years [9]. In the KoBCRAT, we set the age cut-off point at 50 years for two reasons. First, the age-specific breast cancer rate in Korea differs from that in Western populations. In Korea, the age-specific incidence rate of breast cancer increases up to age 49.9; after age 50, the age-specific rate decreases [16]. For model fitting, we established two models based on the age of the patients: <50 years of age and ≥50 years of age. The age-specific incidence rate among Korean women was due to age and cohort effects. This age-specific pattern, which differs from that in Western countries where age-specific rates increase with increases in age, has continued for nearly 20 years [16]–[18]. Second, the mean and median age at menopause among Korean women is 45–49 years [19] which is identical to the peak age of breast cancer incidence. Menopause is an important risk factor in Korea. Menopause had a protective effect on breast cancer, while risk factors such as BMI showed different risk patterns according to a woman's menopausal status [3], [7], [8]. Therefore, we estimated the risk factors separately for two age groups: <50 years and ≥50 years. We also calculated the risk separately according to age.

Among the risk factors included in the KoBCRAT, family history of breast cancer, age at menarche, menopausal status and age at menopause, pregnancy, age at first full-term pregnancy, duration of breast feeding, BMI, and oral contraceptive usage were consistently significant risk factors in previous Korean studies [7], [20]–[25]. Exercise was associated with a lower breast cancer risk in Chinese women [26], and McTieran et al. suggested that 3–4 hours of exercise per week lowered the risk for breast cancer [27]. The inclusion of modifiable factors, such as duration of breast feeding, oral contraceptive intake, BMI and physical activity in the KoBCRAT offers breast cancer risk counselors an intervention method for primary prevention, otherwise the Gail model offers only secondary prevention options for high-risk women, such as targeted screening or chemoprevention.

The Gail model performs well in female populations of the United States. Based on the Gail 2 model, modification of the original Gail model [28], the Breast Cancer Prevention Trial demonstrated a reduction in breast cancer in high-risk women after receiving tamoxifen [29]. However, it is uncertain whether the Gail model performs well in other countries. It has shown good performance in Italian populations [30], [31] but poor performance in Czech and Spanish populations [10], [32]. The major reasons for this inconsistency might be wide variation in breast cancer incidence rates among ethnic groups, leading to various baseline risks, and differences in major risk factors [32]. The age-specific breast cancer incidence rates in Korea peak at the age of 45–49 years and decline thereafter, whereas those in the United States continually increase with age, although the rate of increase differs slightly before and after the age of 50 years. The incidence rates of breast cancer were nearly two-fold higher in the United States than in Korea (76.0 and 39.6/100,000, respectively) [1], [33]. When we applied the original Gail model, which uses the incidence and mortality rate of breast cancer in the US, the estimated 5-year and lifetime risk scores among Koreans became 2- and 4-fold higher than the estimated risk scores determined using the modified Gail model with Gail's parameter estimators and Korean incidence and mortality rates (data not shown). The modified Gail model which applied Korean incidence and mortality data and the parameter estimators from the original Gail model produced lower 5-year risk for the cases than for the controls, while the KoBCRAT produced higher 5-year and lifetime risk for the cases than for the controls. We could not evaluate the effects of the number of prior breast biopsies and biopsy results because they were not measured, and we regarded them as unknowns in the National Cancer Institute Breast Cancer Risk Assessment Macro.

Several breast cancer risk assessment tools have been proposed for Korea. A model with a cohort design and an 8-year follow-up [34] was based on Korea National Health Insurance Corporation claim data (Health Insurance Review and Assessment Service (HIRA) data). The model was internally validated in the same source population using another set of HIRA data, but it had a basic limitation because the source data were secondary data from insurance claims and the model included only three risk factors: age, age at menarche, and breastfeeding duration. Three studies adopted a case-control design [35]–[37], and two of these studies used the subgroup of our study population [35], [36]. The selected risk factors were similar to our results [35], [36]. The other case-control study suggested the calculation of risk scores using meat consumption, past breast disease experience, number of children, family history of breast cancer, and breastfeeding [37]. These three models were not validated in other source populations.

The c-statistics in the KoBCRAT were 0.63 in women aged <50 years and 0.65 in those aged ≥50 years, showing modest discriminative power between breast cancer cases and controls. These values are not inferior to those in the Gail model for Western populations (meta-analyzed c-statistic = 0.63; 95% CI, 0.59–0.67) [38]. When we estimated c-statistics (AUC) of the KoBCRAT in the independent two cohorts, it was marginally significant in the female participants of the KMCC despite small number of incident cases, otherwise higher and significant discriminatory power (c-statistics = 0.89) compared with previous studies [38] in the NCC cohort was presented. This discrepancy between the two cohorts and higher discriminatory power in the NCC cohort may be caused by different population characteristics. The NCC cohort was comprised of participants, aged 30 or over, in a cancer screening program conducted at a teaching hospital located in an urban area; in comparison, the KMCC was comprised of members of rural communities who were between 15 and 85 years old. Considering that the KoBCRAT was developed on the basis of cases from three teaching hospitals located in Seoul, Korea, the higher c-statistics of the NCC cohort despite the small number of incident cases suggests that the discriminatory power of the KoBCRAT was satisfactory for the entire Korean female population—especially women in urban areas.

The E/O ratios of the Gail model for source populations from the United States, United Kingdom, and Italy were 0.75–1.19 [38], whereas those of the KoBCRAT for the KMCC and NCC cohort studies were 0.97 and 0.96, respectively. In both cohort, E/O ratio was near 1 and showed good projection.

Attempts to increase the performance of the Gail model have involved the addition of other risk factors, such as genetic information and breast density; however, the c-statistics were only modestly increased by 0.025 and 0.047 [39], [40]. Among women aged <50 years, the KoBCRAT c-statistic improved slightly for estrogen receptor (ER)+ tumors (0.68) but decreased for ER− tumors (0.64) because most selected risk factors are related to estrogen activity (aromatase production in adipose tissue [41] or ages at menarche and menopause [42]). Therefore, further research is needed to improve discriminating capacity by adding risk factors for ER− tumors and biological risk factors (biomarkers) for breast cancer.

This study has some limitations. The KoBCRAT had moderate discriminatory accuracy although the c-statistic values were similar to those of the Gail model, which might render it inadequate for cancer diagnosis or screening. Therefore, we should design a modified model including other risk factors, such as breast density, bone mineral density, and genetic and molecular biomarker information. Second, previous studies assessed model calibration using E/O ratios according to risk factor or age group categories [15], [43]. However, we could calculate only the overall E/O ratio because a small number of breast cancer cases developed in two independent cohorts. Third, the discriminatory power of the KoBCRAT was better for females living in urban areas; it had only marginally significant power for females living in rural areas. Therefore, efforts to increase the discriminatory power of the KoBCRAT are necessary to cover females living in rural areas or Korean females as a whole. Fourth, the SeBCS cases used in the development of the KoBCRAT were selected from patients enrolled at three teaching hospitals; their disease characteristics could be different from those of breast cancer patients in Korea as a whole. However, when we compared the stage of the patients in the SeBCS with that reported in the Korean Breast Cancer Registry, the distribution was not largely different. For example, the proportion of advanced (stage III or higher) cases was 15.3% among the SeBCS patients and 15.4% among the patients in the Korean Breast Cancer Registry. Thus, the distribution of cases included in the KoBCRAT may be typical of cases in our country. Despite these limitations, the inclusion of modifiable factors, such as breast feeding, oral contraceptive usage, exercise and BMI, in the KoBCRAT allows policymakers to quantify risk reduction after modification and encourages the general population to modify behaviors.

## Conclusions

Considering the rapid increase in breast cancer incidence in recent decades in Korea, the development of breast cancer models targeting the Korean female population is needed. We developed a breast cancer risk assessment tool and conducted validation with two cohorts. The KoBCRAT showed good calibration and modest discrimination, particularly for women living in urban areas or with estrogen receptor-positive tumors. Despite several limitations, the KoBCRAT is more applicable to Korean women than the Gail model based on Western populations. We expect that the KoBCRAT will contribute to future breast cancer clinical trials focused on primary prevention and early detection in Korean women. Further work is needed to increase the discriminatory power of the KoBCRAT for Korean females as a whole.

## Supporting Information

File S1 **Supporting information.** Table S1. Age-specific incidence for breast cancer, breast cancer–specific mortality rate, total mortality rate, and baseline risk of breast cancer in Korea. Table S2. Distribution of basic characteristics of cases and controls. Figure S1. Natural logarithms of the odds ratio for individual relative risk of breast cancer development
(DOC)
