# Supplementary material for: Korean Risk Assessment Model for Breast Cancer Risk Prediction
Source: PLoS One. 2013 Oct 25;8(10):e76736. doi: 10.1371/journal.pone.0076736 (PMC3808381; doi:10.1371/journal.pone.0076736)
Supplement: File S1 — Supporting information. Table S1. Age-specific incidence for breast cancer, breast cancer–specific mortality rate, total mortality rate, and baseline risk of breast cancer in Korea. Table S2. Distribution of basic characteristics of cases and controls. Figure S1. Natural logarithms of the odds ratio for individual relative risk of breast cancer development (DOC) [file pone.0076736.s001.doc]

Table S1. Age-specific incidence for breast cancer, breast cancer–specific mortality rate, total mortality rate, and baseline risk of breast cancer in Korea

| Age group (years) | Incidence rate^a^ | Breast cancer–specific mortality rate^a^ | Total mortality rate^a^ | Baseline risk^a^ |
| --- | --- | --- | --- | --- |
| 15–19 | 0.1 | 0.0 | 22.5 | 0.02649 |
| 20–24 | 1.6 | 0.1 | 37.9 | 0.42390 |
| 25–29 | 7.9 | 0.7 | 52.5 | 2.09299 |
| 30–34 | 27.2 | 2.4 | 62.0 | 6.89831 |
| 35–39 | 57.7 | 5.7 | 73.5 | 14.98037 |
| 40–44 | 98.5 | 9.2 | 94.9 | 27.52837 |
| 45–49 | 137.5 | 13.1 | 134.7 | 43.10345 |
| 50–54 | 124.3 | 15.8 | 189.0 | 32.51880 |
| 55–59 | 105.4 | 17.7 | 268.9 | 31.58313 |
| 60–64 | 95.2 | 16.7 | 420.0 | 29.53947 |
| 65–69 | 69.9 | 16.0 | 729.5 | 21.61802 |
| 70–74 | 60.8 | 14.2 | 1,406.3 | 19.49483 |
| 75–70 | 40.8 | 14.4 | 2,821.0 | 13.08206 |
| 80–84 | 35.6 | 19.8 | 5,495.8 | 11.41473 |
| 85–89 | 25.9 | 26.7 | 10,052.3 | 8.30454 |

^a^ per 100,000.

Table S2. Distribution of basic characteristics of cases and controls

|  | | | Cases | | Controls | | P-value |
| --- | --- | --- | --- | --- | --- | --- | --- |
|  | | | N | (%) | N | (%) |  |
| **Family history of breast cancer in first-degree relatives** | | | | |  |  |  |
|  | No | | 3601 | (95.0) | 3688 | (97.3) | <0.001 |
|  | Yes | | 188 | (5.0) | 101 | (2.7) |  |
| **Age at menarche (years)** | | |  |  |  |  |  |
|  | | <13 | 218 | (5.8) | 186 | (5.1) | <0.01 |
|  | | 13-16 | 2821 | (75.5) | 2632 | (71.6) |  |
|  | | ≥17 | 700 | (18.7) | 858 | (23.3) |  |
| **Menopause** | | |  |  |  |  |  |
|  | | Premenopausal | 2238 | (59.5) | 2016 | (54.2) | <0.001 |
|  | | Postmenopausal | 1523 | (40.5) | 1707 | (45.8) |  |
| **Age at menopause (years)** | | |  |  |  |  |  |
|  | | Premenopausal | 2238 | (60.7) | 2016 | (55.8) | <0.001 |
|  | | <44 | 236 | (6.4) | 268 | ((7.4) |  |
|  | | 45–49 | 452 | (12.3) | 548 | (15.2) |  |
|  | | 50-54 | 633 | (17.2) | 664 | (18.4) |  |
|  | | ≥55 | 130 | (3.5) | 114 | (3.2) |  |
| **Pregnancy** | | |  |  |  |  |  |
|  | | Nullipara | 308 | (8.2) | 311 | (8.3) | 0.900 |
|  | | Para | 3481 | (91.9) | 3478 | (91.8) |  |
| **Age at first full-term pregnancy (years)** | | | |  |  |  |  |
|  | | Nullipara | 308 | (8.2) | 311 | (8.3) | <0.001 |
|  | | <24 | 846 | (22.4) | 979 | (26.3) |  |
|  | | 24–30 | 2347 | (62.2) | 2243 | (60.2) |  |
|  | | ≥31 | 273 | (7.2) | 195 | (5.2) |  |
| **Duration of Breast feeding (months)** | | | | | | |  |
|  | | Never | 1024 | (29.7) | 940 | (39.4) | <0.001 |
|  | | 0~6 | 561 | (16.3) | 282 | (11.8) |  |
|  | | >6 | 1857 | (54.0) | 1162 | (48.7) |  |
| **Body mass index** | | |  |  |  |  |  |
|  | | <25 | 2825 | (75.4) | 2788 | (75.4) | 0.068 |
|  | | 25–29.9 | 811 | (21.6) | 829 | (22.4) |  |
|  | | ≥30 | 113 | (3.0) | 81 | (2.2) |  |
| **Oral contraceptive usage** | | |  |  |  |  |  |
|  | | Never | 3000 | (91.1) | 1831 | (93.0) | 0.014 |
|  | | Ever | 292 | (8.9) | 137 | (7.0) |  |
| **Exercise** | | |  |  |  |  |  |
|  | | <once/week | 2003 | (73.2) | 1102 | (65.2) | <0.001 |
|  | | ≥once/week | 733 | (26.8) | 588 | (34.8) |  |

Figure S1. Natural logarithms of the odds ratio for individual relative risk of breast cancer development

| The Korean Breast Cancer Risk Assessment Tool for women aged < 50 years  = -1.2700 + 0.1136 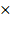 (breast cancer history in first-degree relatives: code 1 if yes, code 0 if no) + 0.6224 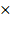 (age at menarche: code 1 if <13 years, code 0 if others) + 0.3624 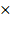 (age at menarche: code 1 if 13-16 years, code 0 if others) + 0.5539 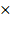 (menopausal status: code 1 if premenopausal, code 0 if postmenopausal) + 0.0763 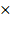 (age at first full-term pregnancy: code 1 if nullipara, code 0 if others) + 0.1451 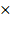 (age at first full-term pregnancy: code 1 if 24–30 years, code 0 if others) + 0.2264 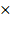 (age at first full-term pregnancy: code 1 if ≥ 30 years old, code 0 if others) + (-0.0722) 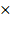 (duration of breast feeding: code 1 if never, code 0 if others) + 0.2198 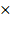 (duration of breast feeding: code 1 if 0-6 months, code 0 if others) + 0.2121 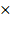 (oral contraceptive usage: code 1 if yes, code 0 if never) + 0.2878 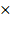 (exercise: code 1 if <once/week, code 0 if ≥once/week)  The Korean Breast Cancer Risk Assessment Tool (KoBCRAT) for women aged ≥ 50 years  = -0.6729 + 0.6960 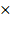 (breast cancer history in first-degree relatives: code 1 if yes, code 0 if no) + 0.8755 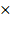 (age at menarche: code 1 if <13 years, code 0 if others) + 0.4244 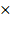 (age at menarche: code 1 if 13-16 years, code 0 if others) + 0.9154 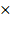 (age at menopause: code 1 if premenopausal, code 0 if others) + 0.2954 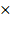 (age at menopause: code 1 if 45-49 years, code 0 if others) + 0.3048 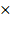 (age at menopause: code 1 if 50-54 years, code 0 if others) + 0.4794 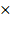 (age at menopause: code 1 if ≥ 55 years old, code 0 if others) + 0.6287 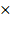 (Pregnancy: code 1 if nullipara, code 0 if para) + 0.1474 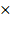 (body mass index: code 1 if 25.0–29.9, code 0 if others) + 0.8239 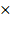 (body mass index: code 1 if ≥30, code 0 if <30) + 0.4175 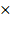 (oral contraceptive usage: code 1 if once a week or more, code 0 if less than once a week) + 0.6115 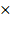 (exercise: code 1 if <once/week, code 0 if ≥once/week)” |
| --- |
